# Beta Adrenergic Signaling: A Targetable Regulator of Angiosarcoma and Hemangiosarcoma

**DOI:** 10.3390/vetsci2030270

**Published:** 2015-09-21

**Authors:** Erin B. Dickerson, Brad A. Bryan

**Affiliations:** 1 Department of Veterinary Clinical Sciences, University of Minnesota College of Veterinary Medicine, St. Paul, MN 55108, USA; 2 Animal Cancer Care and Research Program, University of Minnesota, St. Paul, MN 55108, USA; 3 Masonic Cancer Center, University of Minnesota, Minneapolis, MN 55455, USA; 4 Department of Biomedical Sciences, Texas Tech University Health Sciences Center, El Paso, TX 79912, USA; E-Mail: brad.bryan@ttuhsc.edu; 5 Paul L. Foster School of Medicine, Texas Tech University Health Sciences Center, El Paso, TX 79912, USA

**Keywords:** angiosarcoma, beta adrenergic receptor, canine, CXCL12, CXCR4, hemangiosarcoma, S1P_1_, uPAR, vascular sarcoma

## Abstract

Human angiosarcomas and canine hemangiosarcomas are highly aggressive cancers thought to arise from cells of vascular origin. The pathological features, morphological organization, and clinical behavior of canine hemangiosarcomas are virtually indistinct from those of human angiosarcomas. Overall survival with current standard-of-care approaches remains dismal for both humans and dogs, and each is likely to succumb to their disease within a short duration. While angiosarcomas in humans are extremely rare, limiting their study and treatment options, canine hemangiosarcomas occur frequently. Therefore, studies of these sarcomas in dogs can be used to advance treatment approaches for both patient groups. Emerging data suggest that angiosarcomas and hemangiosarcomas utilize beta adrenergic signaling to drive their progression by regulating the tumor cell niche and fine-tuning cellular responses within the tumor microenvironment. These discoveries indicate that inhibition of beta adrenergic signaling could serve as an Achilles heel for these tumors and emphasize the need to design therapeutic strategies that target tumor cell and stromal cell constituents. In this review, we summarize recent discoveries and present new hypotheses regarding the roles of beta adrenergic signaling in angiosarcomas and hemangiosarcomas. Because the use of beta adrenergic receptor antagonists is well established in human and veterinary medicine, beta blockade could provide an immediate adjunct therapy for treatment along with a tangible opportunity to improve upon the outcomes of both humans and dogs with these diseases.

## 1. Introduction

Angiosarcomas represent a subgroup (approximately 2%–3%) of soft tissue sarcomas, characterized by both their aggressive behavior and clinical heterogeneity [1,2,3]. While these tumors are thought to originate from blood vessel-forming cells, our understanding of the pathology of angiosarcomas remains limited. The infrequent diagnosis of angiosarcomas in humans adds a further challenge to the development of effective therapies. The most common site of involvement is the head and neck region, accounting for approximately 50% of all cases [4,5]. Visceral angiosarcomas arise in the right atrium, liver, spleen, or lung, while cutaneous or subcutaneous angiosarcomas arise from skin, soft tissues, or the mammary gland, and hematogenous angiosarcomas can arise from bone or bone marrow [6]. More than half of the patients with this disease die within their first year of diagnosis, and the 5-year survival rate, when all anatomical sites are combined, hovers around 30% [4]. It is generally established that patients with angiosarcomas of the head and neck, the breast, and extremities demonstrate longer overall survival than those with angiosarcomas of visceral sites such as the spleen and heart [7,8]. Exposure to genotoxic agents such as vinyl chloride, thorium, and arsenic, as well as radiation therapy (mainly secondary angiosarcomas in women who have undergone treatment for breast cancer), have been implicated in the etiology of some angiosarcomas, but most patients have no history of exposure to these risk factors, leaving the cause of their disease unknown [6,9].

Hemangiosarcomas in dogs arise spontaneously and occur commonly [10]. Hemangiosarcomas are most often associated with viscera, primarily the spleen and right atrium, but they can also occur in skin, bone, and virtually every other tissue [11]. Cutaneous hemangiosarcomas tend to be confined regionally, although they can be locally invasive, and are amenable to surgery and adjuvant chemotherapy. As in humans, visceral hemangiosarcomas are more aggressive and demonstrate poor survival [4,12]. The expected survival for dogs with splenic hemangiosarcomas (the most common form) treated with standard of care surgery and with adjuvant chemotherapy typically ranges from three to six months [11]. Dogs where the disease is confined to an organ without evidence of bleeding or tumor rupture (Stage I) and some dogs with splenic rupture with or without regional metastasis (Stage II) (approximately 15% of all cases) at diagnosis can be expected to survive up to or beyond 12 months [11]. Hemangiosarcoma has an alarmingly high predilection for some breeds, such as Golden Retrievers, Portuguese Water Dogs, Boxers, and German Shepherd Dogs [13,14,15], providing evidence that genetic background plays an important role in predisposed susceptibility to some hemangiosarcomas. However, as in humans, the general underlying causes of the disease in dogs remain obscure. For a more complete review of the history and pathobiology of canine hemangiosarcoma, the reader is referred to the review by Kim *et al.* within this series [16].

Surgery, in combination with chemotherapy, has served as the cornerstone for treatment of both humans and dogs with locally advanced or metastatic disease. In humans, recent studies have shown that doxorubicin-based regimens and weekly paclitaxel have similar efficacy [17,18], while cutaneous angiosarcomas respond more favorably to a weekly paclitaxel regimen [17]. In dogs, doxorubicin-based therapies have generally provided the most improved survival times [11]; however, the overall response rate to conventional therapy remains poor. Recent reports show conflicting data regarding the potential benefit of adding dacarbazine to conventional doxorubicin-based protocols, and resolution of this issue will require further study [19,20]. Alternative approaches to conventional single agent chemotherapy protocols, including combinations of cytotoxic drugs [19,21,22,23,24,25,26,27,28,29], metronomic dosing [30], autologous vaccines [31], and immune-based and antiangiogenic therapies [32] have also been studied in dogs. None of these have shown benefit over the current standard-of-care, leaving both human and canine patients to confront what is likely to be a bleak prognosis.

Recent data from our group and others show progress and the promise in defining the cell signaling mechanisms used by angiosarcomas and hemangiosarcomas for proliferation, tumor growth, and drug resistance. In this review, we will present recent data suggesting that angiosarcomas and hemangiosarcomas utilize beta adrenergic signaling to drive many of these recently identified pathways, fueling tumor progression. We will also discuss how these pathways can be disrupted by beta blockade for therapeutic intervention.

## 2. Beta Adrenergic Signaling and Cancer

### 2.1. Inhibition of Beta Adrenergic Signaling and Therapeutic Responses

Beta adrenergic receptor (β-AR) antagonists (beta blockers) are a commonly prescribed group of drugs used to treat hypertension, heart failure, arrhythmias, and anxiety. Growing epidemiological evidence over the past several years has revealed a strong correlation between reduced progression, metastasis, and mortality, and the use of beta blockers in cancer patients [33,34,35,36,37,38,39,40]. This has led to the initiation of clinical phase II studies evaluating the effect of the beta blocker propranolol in ovarian, cervical, colorectal, breast, and prostate cancers, as well as investigation of the effects in melanoma and solid tumors (ClinicalTrials.gov). Because beta blockers are clinically well characterized in human and veterinary medicine and have safely been administered as therapeutics for cardiovascular disease, they could provide novel agents that can be exploited clinically for cancer treatment. Similar retrospective studies have not yet been performed in veterinary medicine, but the strong parallels between some cancers in humans and companion animals [41,42,43,44] suggest that beta blockade may provide similar benefits in the treatment of certain cancers.

The beta adrenergic signaling pathway is part of the sympathetic nervous system, which mediates the fight-or-flight response to ensure that the body responds quickly to physical danger or a perceived threat. Fibers of the sympathetic nervous system innervate most major organ systems. In response to stress, the catecholamine, norepinephrine, is released locally by sympathetic nerve endings into target tissues and systematically from the adrenal medulla into the bloodstream. Levels in the blood are also elevated through the release of epinephrine from the adrenal medulla. The biological effects of norepinephrine and epinephrine are mediated by alpha1-, alpha2-, and beta-adrenergic receptor families, which show distinct patterns of tissue distribution and signal through distinct biochemical pathways [45,46]. At the cellular level, many of the pathways involved in adrenergic receptor signaling regulate apoptosis, proliferation, and angiogenesis in normal tissues. Therefore, it is not surprising that different cancer types have been found to express functional adrenergic receptors, which regulate many of the processes for tumor growth. The three subtypes of beta adrenergic receptors, β1-AR, β2-AR, and β3-AR are present at many sites of tumor growth and metastasis including pancreatic [47], lung [48], breast cancer [49], melanoma [50], prostate cancer [51], and neuroblastoma [52]. Beta adrenergic receptor expression has also been confirmed in Ewing’s sarcoma [52]. We recently identified the expression the three receptor subtypes in angiosarcoma [53,54] and hemangiosarcoma [54], implicating beta adrenergic signaling in the progression of these tumors and supporting the investigation of beta blockers as a novel treatment approach.

### 2.2. Beta Adrenergic Signaling and the Regulation of Tumor Cell Biology

**Figure 1:**
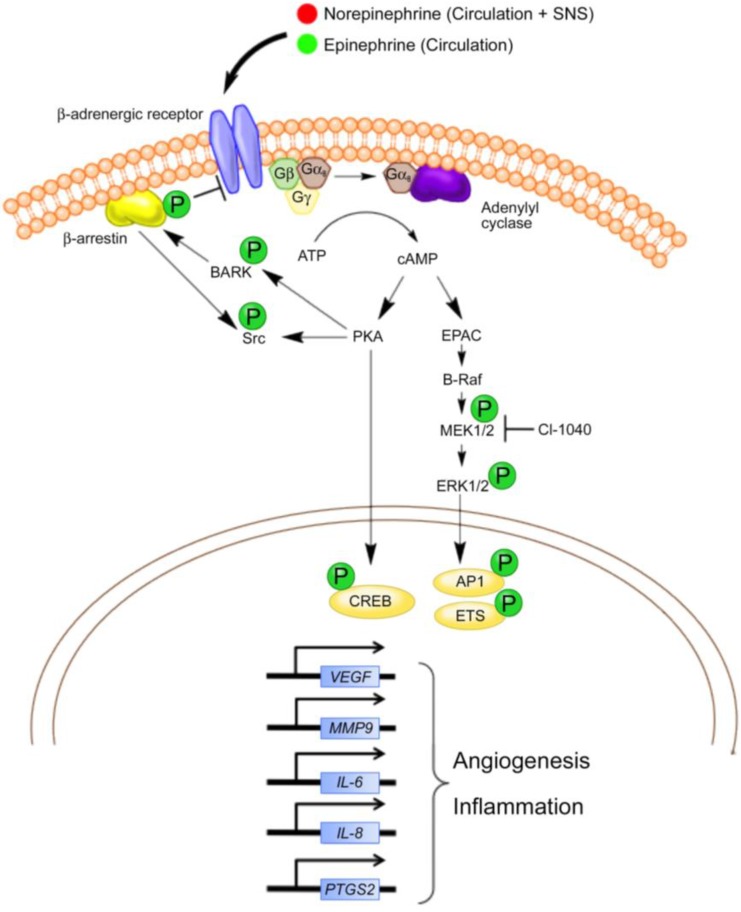
Summary of potential beta adrenergic signaling pathways in canine hemangiosarcoma and human angiosarcoma. Although several other pathways and transcription factors are regulated through beta adrenergic signaling, pathways currently described or thought to be active in hemangiosarcoma and angiosarcoma cells are presented. Norepinephrine and epinephrine are delivered into the tumor microenvironment through the circulation and potentially from local sympathetic nerve fibers. The catecholamines bind to β-ARs, causing G_αs_-mediated activation of adenylyl cyclase and the generation of cAMP from ATP. Intracellular cAMP activates two major biochemical signaling pathways (**1**) protein kinase A (PKA) and (**2**) exchange protein activated by adenylyl cyclase (EPAC). Activation of PKA leads to the phosphorylation of multiple protein targets, such as transcription factors (e.g., CREB) and the beta adrenergic receptor kinase (BARK). BARK recruits β-arrestin, which inhibits beta adrenergic signaling and activates Src kinase. Although not shown, Src kinase is known to activate other transcription factors (e.g., STAT3) and the downstream focal adhesion kinase (FAK) in other cancers. The activation of EPAC leads to the activation of the B-Raf/ mitogen-activated protein kinase (MAPK) signaling pathway and affects the AP1 and Ets family of transcription factors. In general, the transcriptional responses potentially attributed to β-AR activation in angiosarcomas and hemangiosarcomas include the upregulation of genes involved in angiogenesis and inflammation. Adapted with permission from [57].

Here, we provide a general overview of beta adrenergic signaling in the context of angiosarcoma and hemangiosarcoma biology. For more details on this topic, the reader is referred to several recent reviews [55,56,57,58,59]. In the classical signaling pathway, norepinephrine and epinephrine from the circulation bind to beta adrenergic receptors as G protein-coupled receptors and activate the adenylyl cyclase/cAMP/protein kinase A (PKA) signaling cascade (Figure 1). This signaling pathway regulates a wide range of cellular processes such as metabolism and growth as well as more cell-specific processes such as gene transcription, differentiation, motility, and morphology. In turn, PKA induces the phosphorylation of multiple transcription factors, including members of the cAMP response element bind protein (CREB) family. Phosphorylation of CREB promotes its binding to the cAMP response element (CRE) of certain genes, inducing the transcription of several proangiogenic factors, including vascular endothelial growth factor (VEGF), matrix metalloproteinase (MMP)-9, and interleukin (IL)-6. These factors are key modulators of angiogenesis and inflammation, which are recurrent features of hemangiosarcomas [60,61]. VEGF, MMP-9, and IL-6 are highly expressed by hemangiosarcoma tumor cells, suggesting that hemangiosarcomas use these factors to alter their tumor microenvironment (TME) and promote tumor growth. PKA has also been shown to activate Src directly in ovarian cancer cells in response to beta adrenergic signaling [62]. We previously described the activation of Src in canine hemangiosarcoma cell lines [63]. Inhibition of this signaling process through the tyrosine kinase inhibitor dasatinib was detrimental to hemangiosarcoma cell viability. We have not yet determined if Src activation is regulated beta adrenergic signaling in angiosarcomas/hemangiosarcomas.

Signaling through β-ARs can also be independent of PKA signal transduction. A frequently cited example is the exchange protein activated by cAMP (EPAC) mediated signal transduction cascade [64]. EPAC stimulates the downstream effectors B-Raf, MEK, and MAPK, which stimulate cell growth and proliferation. Recent studies by Andersen *et al*. [65] showed that a MEK inhibitor, CI-1040, reduced the activation of MAPK family member ERK as well as the viability of primary cells derived from canine hemangiosarcomas. Moreover, CI-1040 decreased the growth of cutaneous cell-derived xenografts. Positive staining for activated ERK1/2 was noted in about 50% of archived human angiosarcoma samples, confirming the idea that the same pathways are activated in human anigosarcoma and supporting a role for MAPK signaling in tumor growth. Although some overlap occurs between the PKA and EPAC signaling mechanisms, the influence of beta adrenergic signaling on angiogenesis and inflammation seems to be mediated predominately by PKA induction of genes encoding the growth factors and cytokines involved in these processes.

## 3. Inhibition of Beta Adrenergic Signaling in Vascular Tumors

### 3.1. Beta Blockade of Benign Vascular Tumors: Studies in Infantile Hemangiomas Point the Way

The remarkable efficacy of beta blockers against vascular tumors was first identified in infantile hemangiomas (IH), and this serendipitous finding has revolutionized the management of IHs to become the standard of care [66]. IHs are the most common benign tumors in infancy affecting 4%–10% of the population. The tumors are largely composed of densely packed, over-proliferating capillaries with high cellular density and the absence of an open lumen. Lesions show a predilection for female infants at a rate three times that of males, and low birth weight as well a premature birth also seem to be contributing factors [67,68]. The head and neck region is the most frequently involved area (60%), followed by the trunk (25%), and the extremities (15%). IHs have a predictable natural history, arising soon after birth and progress through three defined stages: (a) endothelial proliferation without defined vasculature (b) involution, where blood vessel formation becomes defined; and (c) replacement of blood vessels with a fibrofatty residuum, which often leads to spontaneous regression of the tumor. Because the majority of these tumors regress spontaneously, most children with IHs require no treatment. However, approximately 10% of cases, depending on their anatomical site and/or size, can be serious or life threatening complications requiring immediate intervention. Propranolol is currently the preferred treatment for problematic, proliferating IH [69], but the optimal regimen dosage and treatment duration remain unknown. Because side effects (e.g., hypotension, bradycardia, hypoglycemia, *etc.*) can occur in up to 60% of cases [70], lower dosages that accelerate involution of IH are being used; however, treatment needs to continue for approximately 14 months to avoid regrowth [69,71].

### 3.2. Beta Blockade of Malignant Vascular Tumors

The ability of beta blockade to reduce the size and appearance of IHs [72,73], along with the discovery of β-AR expression by angiosarcomas and hemangiosarcomas, suggested that malignant vascular tumors would also be susceptible to beta blockade. Recent work demonstrated that propranolol effectively reduced proliferation and induced apoptosis in a panel of angiosarcoma cell lines [54]. Moreover, propranolol inhibited angiosarcoma growth in a xenograft tumor model and enhanced the effect of standard chemotherapy agents to promote cell death. Mechanistically, propranolol altered the levels of key cell cycle regulators Cdk4, Cdk6, cyclin D1, and cyclin E1, an effect noted previously in IH endothelial cells treated with isoprenaline and β2-AR antagonists, suggesting shared growth mechanisms between the benign and malignant vascular tumors. Potent upregulation of the cell cycle inhibitors, p21 and p27, was also observed.

The results of these studies have translated successfully to the clinic where we recently reported the treatment of a male patient with a beta adrenergic positive cutaneous angiosarcoma involving 80% of the scalp, left forehead, and left cheek with no evidence of metastasis [53]. Treatment with propranolol for one week decreased the proliferative index of the tumor (~34%), providing evidence that propranolol alone could inhibit tumor growth. A combination of propranolol, chemotherapy, and radiation over an eight-month period resulted in extensive tumor regression with no detectable metastases. During the course of this study, Banavali *et al.* [74] reported that the combination of propranolol and two cycles of metronomic chemotherapy over a six month period resulted in a complete response of a relapsing metastatic angiosarcoma on the upper extremity of a patient. While the study by Banavali *et al.* further supports the use of beta blockers for these tumors, their experimental setup could not distinguish the contribution of propranolol from the contribution of metronomic chemotherapy toward tumor relapse. At this time, no assumptions regarding overall patient survival can be made from these studies, and more studies with expanded patient populations receiving single agent therapy (beta blockade) or combination therapy will be necessary to fully evaluate the adjuvant nature of propranolol. These data also support the investigation of beta blockade for the treatment of canine hemangiosarcoma. The establishment of clinical trials in pet dogs is likely to have a greater margin of success due to the high incidence of hemangiosarcomas within the pet dog population. Furthermore, the continued documentation of similarities between the human and canine diseases suggests that results from clinical trials in dogs are likely to be informative for the treatment of angiosarcoma in humans [75].

## 4. Beta Adrenergic Regulation of the Hematopoietic and Tumor Cell Microenvironments

**Figure 2:**
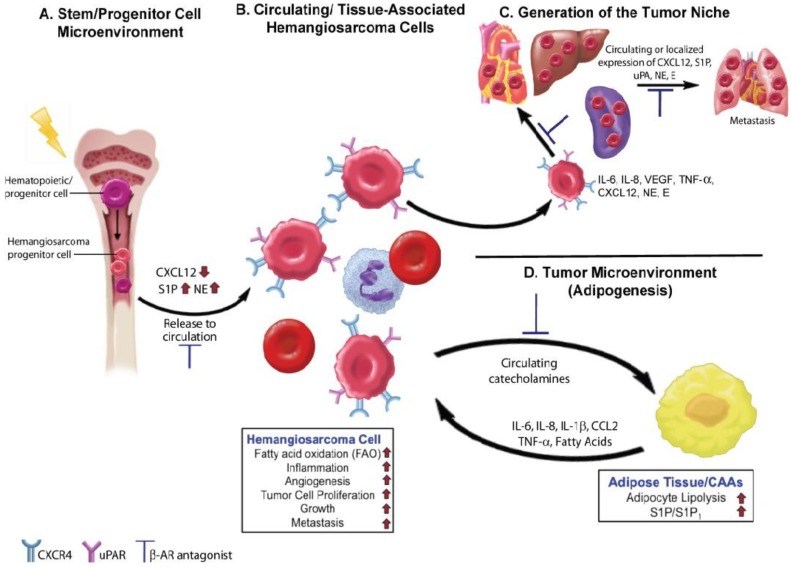
Beta adrenergic signaling drives essential processes in tumor initiation and progression in canine hemangiosarcomas and human angiosarcomas. (**A**) Norepinephrine (NE) is released by the sympathetic nervous system in the bone marrow, where it binds to β-AR and causes a decrease in the release of CXCL12 within hematopoietic stem cell niches. Reductions in CXCL12 levels lead to increased hematopoiesis. In hemangiosarcomas, DNA damaging events may occur at the level of hematopoietic stem or progenitor cells leading to the generation of hemangiosarcoma progenitor cells. Increased levels of NE, might then promote the release of hemangiosarcoma cells into the circulation. Inhibition of NE activity by β-AR antagonists could limit disease progression by restoring CXCL12 levels and regulation of the bone marrow microenvironment; (**B**) Hemangiosarcoma cells released into the circulation may exist as progenitor cells or more differentiated tumor cells. The hemangiosarcoma cells express CXCR4 receptors, which promote tumor cell homing to tissue sites that express high levels of CXCL12. S1P may also be produced by RBCs and platelets in the circulation and in tissues to further promote homing and tumor cell dissemination; (**C**) The secretion of proangiogenic and proinflammatory factors by hemangiosarcoma cells promotes the formation of the tumor cell niche. NE and epinephrine (E) released locally by sympathetic nerve fibers or found circulating in the blood may further activate tumor cells and promote tumor initiation and growth; (**D**) Tumor cells possess the ability to hijack the metabolic processes of normal cells, such as adipocytes, present within the surrounding local environment. Activation of β-AR on adipocytes by locally produced or circulating NE and E can lead to the further production and release of angiogenic and inflammatory factors, promoting tumor growth. Upon beta adrenergic stimulation, adipocytes release fatty acids into the tumor microenvironment, which can then serve as a metabolic fuel for tumor cells or for the production of essential metabolites needed for efficient tumorigenesis. Potential areas in this process where β-AR antagonists may slow or halt tumorigenesis are indicated.

Accumulating evidence suggests that hemangiosarcomas originate from hematopoietic progenitor cells (HPCs) that retain multipotency and can give rise to tumors with angiogenic, inflammatory, and adipogenic signatures [60,61,76]. The potential hematopoietic origin of this disease implicates β-ARs as key regulators of hemangiosarcoma progression since signaling through these receptors regulates the egress of hematopoietic stem cells (HSCs) from the bone marrow microenvironment (Figure 2A) [77,78]. Moreover, the presence of the identified angiogenic, inflammatory, and adipogenic signatures from primary hemangiosarcomas (Figure 2B) [60] and in hemangiosarcoma cell lines and tumor progenitor cells identified from these lines [60,61,76] suggest that hemangiosarcoma cells are capable of modulating the TME (Figure 2C,D). Because beta adrenergic signaling regulates the functions of many cell types relevant to cancer progression and metastasis, including vascular pericytes, adipocytes, fibroblasts, and immune cells [45,46,58], and beta adrenergic signaling pathways impact many aspects of cancer progression, including angiogenesis, inflammation, and lipid metabolism, [58,59], we now suspect much of the coordinated signaling between tumor cells and the TME is regulated through β-ARs. Here, we will highlight the roles of beta adrenergic signaling in the hematopoietic cell niche and the TME. We will then present these roles in the context of recent findings regarding the mechanisms and signaling pathways found in canine hemangiosarcomas. Finally, we will present the novel hypothesis that the recently reported adipogenic signature [60] partially reflects the lipid metabolic preferences of angiosarcomas and hemangiosarcomas, and that these processes may be disrupted by beta blockade.

### 4.1. The β-AR-CXCR4-CXCL12 Signaling Axis

The proliferation and differentiation of HSCs in the bone marrow is followed by the release of hematopoietic stem and progenitor cells (HSPCs) into the circulation. This process is regulated by dynamic interactions between the immune and nervous systems with the stromal environment of the bone marrow niche. One of the most explored HSC niche interactions is between the chemokine receptor CXCR4 and its ligand, stromal derived factor 1α (SDF-1α) or CXCL12. CXCL12 is a potent chemoattractant for the retention of human and murine HSCs in the bone marrow [79,80], and CXCL12 and CXCR4 are highly expressed by stromal cells within specialized bone marrow niches [80,81]. On the other hand, norepinephrine released from the sympathetic nervous system signals through β-ARs in bone marrow niche cells to decrease CXCL12 levels [78,82]. Consequently, HSC proliferation is elevated, leading to an increased output of immune cells into the circulatory system. In hemangiosarcoma, the heterogeneous expression of CXCR4 and CXCL12 was reported in hemangiosarcoma cell lines and whole tumor tissues by transcriptome analysis [83]. *In vitro*, CXCL12 promoted cell migration and invasion of hemangiosarcoma cells, and these responses were also sensitive to the CXCR4 antagonist, AMD3100. These findings suggest CXCL12 potentiates the migration and invasion of canine hemangiosarcoma cells through CXCR4 signaling. Considering the proposed bone marrow origin of hemangiosarcoma [76], hemangiosarcoma cells expressing CXCR4 on their cell surface may represent a progenitor population that is maintained by CXCL12-abundant reticular cells in this niche [81,84]. The potential also exists that CXCR4 and CXCL12 expression may be regulated through beta adrenergic signaling, and beta blockade may prevent the expansion of hemangiosarcoma progenitor cells from the bone marrow or the migration of these cells to CXCL12-rich sites, reducing metastatic spread.

The importance of β-AR-CXCR4-CXCL12 signaling as a key mediator of hemangiosarcoma progression is corroborated further by the identification of the spingosine-1-phosphate (S1P)/spingosine-1-phosphate receptor 1 (S1P_1_) [85] and the urokinase-type plasminogen activator (uPA)/urokinase-type plasminogen activator receptor (uPAR) [86] signaling axes within these tumors. Growing evidence indicates that both pathways are mediated through CXCR4/CXCL12 signaling [87,88], suggesting they may also be regulated directly or indirectly through β-ARs. Activation of the β3-AR has also been shown to increase the production of S1P in adipocytes [89], and S1P has recently emerged as a novel chemoattractant for HSPCs. The S1P receptors are expressed on both the stem and stromal cells of the bone marrow, and the concentrations of CXCL12 in the bone marrow and S1P in the bloodstream dictate the direction of stem cell movement into and out the bone marrow and the circulation [90,91]. S1P is also highly produced by mature red blood cells [92], activated platelets [93], as well as endothelial cells [94,95,96], which were recently identified as a major source for circulating S1P. Microarray data comparing genome-wide gene expression from canine hemangiosarcoma cell lines and non-malignant endothelial cells showed that S1P_1_ is highly expressed in hemangiosarcoma cell lines and primary hemangiosarcomas [85]. S1P also increased the proliferation of hemangiosarcoma cell lines and prolonged exposure of these cells to FTY720 (a S1P_1_ agonist and antagonist) decreased S1P_1_ expression and induced apoptosis. Although hemangiosarcoma cells produced little or no S1P, they selectively consumed S1P from the cell culture medium, suggesting that S1P may play a role in the metastasis of hemangiosarcomas. A similar response was reported in human rhabdomyosarcoma cells wherein S1P strongly enhanced *in vitro* motility [97]. In this same study, the levels of S1P were increased in several organs after chemotherapy, suggesting that chemotherapy and the induction of S1P expression may create an unwanted prometastatic environment [97]. It is interesting to speculate that a similar mechanism may contribute to the metastatic progression observed in hemangiosarcomas and angiosarcomas.

Recent studies have shown that the uPA/uPAR signaling axis can also be modulated by CXCL12 [87] and S1P [98]. Like S1P, uPA/uPAR signaling has been implicated in HSPC mobilization and proliferation [99,100,101] as well as invasion of cancer cells, mesenchymal stem cells, and immune cells [98,102,103]. The identification of uPA/uPAR signaling in hemangiosarcomas has led to the development of a novel therapeutic approach targeting uPAR. This strategy uses a genetically engineered, bispecific ligand targeted toxin (BST) that kills hemangiosarcoma tumor cells as well as an identified progenitor cell population with high specificity [86]. The BST consists of epidermal growth factor (EGF) and uPA (EGFuPA) on the same molecule with truncated Pseudomonas exotoxin. The toxin was mutated to remove amino acids recognized by B-cells, and it was designed to simultaneously target EGF receptors (EGFR) and/or uPAR, which may be expressed by tumor cells and cells in the TME. Based on the *in vitro* results and the efficacy and documentation of safety in laboratory models, a clinical trial (SRCBST) to extend this therapy to dogs with spontaneous hemangiosarcoma was designed. The initial results have shown promise with the overall survival of 56% at 400 days for dogs given an optimal dose of the drug compared to the survival of an historical control group at 7% [104]. The significant biological activity of the drug supports further studies aimed at disrupting the uPA/uPAR signaling axis, and more specifically, the consideration of EGFuPA for a phase 1 study for drug-refractory sarcomas in human patients.

### 4.2. Angiogenesis and Inflammation

The ability of epinephrine and norepinephrine to increase the expression of the angiogenic factor, VEGF, and induce angiogenesis and tumor growth is documented in a variety of cancer types [105,106,107,108,109]. In conjunction with the increased expression of VEGF and MMPs [109], other angiogenic and inflammatory proteins such as IL-6 and IL-8 are also elevated by catecholamine signaling [108,110,111]. It is thought that these molecules are the major initiators of angiogenesis and metastasis controlled by beta adrenergic signaling. More specifically, signaling through β2-AR has been shown to play a key role in the control of angiogenesis. This role was clearly demonstrated by studies of adenoviral-mediated endothelial overexpression of the receptor in a model of hindlimb chronic ischemia [112]. Increased β2-AR expression improved angiographic blood flow and hindlimb perfusion. Angiogenesis was also severely impaired in β2-AR-/- mice subjected to femoral artery resection; angiogenesis was restored in this model by adenoviral-mediated gene transfer of the human β2-AR [113], clearly defining the role of this receptor in regulating angiogenesis. Given the angiogenic signature identified in canine hemangiosarcomas [60,61], β2-AR is likely to play a predominant role in the signaling and progression of these tumors. Our own data support this premise since these tumors respond to beta blockade by propranolol [54], a β1-AR and β2-AR selective antagonist.

While the direct effect of catecholamines on tumor cells is evident, the role of the sympathetic nervous system within the construction of the TME has come to the forefront in several recent studies. Magnon *et al.* [114] demonstrated that the sympathetic nervous system plays a role in establishing the tumor niche for cancer cell survival, while signaling through the parasympathetic nervous system was found to be important for the later stages of tumor growth. They also identified a positive clinical correlation between the infiltration of the TME by nerve fibers and the poorer prognosis of prostate cancer patients. In melanoma, expression of the β3-AR has been shown to correlate with melanoma aggressiveness [115]. While expression of all three β-ARs in primary melanomas has been confirmed, expression of the β3-AR was also documented in stromal, inflammatory, and vascular cells of the melanoma TME. Furthermore, β3-AR activation in these cells was shown to drive angiogenesis and melanoma aggressiveness. These findings demonstrate that the effects of neurotransmitters on tumor cells and on the surrounding stromal cells interact to promote tumor initiation and growth and support the use of β-AR antagonists as an emerging strategy to disrupt these interactions.

Angiogenesis and inflammation are also recurrent features of hemangiosarcomas [60,61]. The tumor cells express functional receptors for growth factors, cytokines, chemokines, and other molecules involved in the angiogenic and inflammatory processes including VEGF, IL-6, IL-8, CXCL12, and PTGS2 (COX-2) [60,61,116]. The inability to define the contributions of “tumor” *vs.* “stroma” remains a challenge in sarcomas due to the integral contribution of stromal cells to the overall tumor organization; however, new strategies are being used to make inroads into this process. Through the generation and characterization of hemangiosarcoma cell lines, several groups have begun to identify the molecular networks in the tumor cells that contribute to the pathogenesis of this disease [60,61,76,116,117,118,119]. The use of cell lines in combination with genome-wide gene expression analysis has provided a new tool to explore the networks of the TME [60]. In this context, we recently documented the direct correlation between IL-8 expression and the reactive TME, showing active inflammation, fibrosis, and coagulation in tumor tissues and cell lines and indicating that hemangiosarcoma cells modulate their TME. The importance of IL-8 in the formation of a tumor niche was further illustrated by the observation that anti-IL-8 antibodies inhibited tumor cell survival and engraftment in a mouse xenograft model [120]. While the role of adrenergic signaling in this process remains to be confirmed, combining xenograft models with *in situ* gene signatures from canine tumors and from cell lines will allow us to test predictions about mechanisms that are operative during the early, and potentially later stages, of tumorigenesis in hemangiosarcomas. This approach will also allow us to make predictions about the roles of β-ARs and adrenergic signaling in tumor and stromal cell interactions within the TME.

### 4.3. Lipid Metabolism

While the influence of catecholamines and beta adrenergic signaling on cancer cell proliferation, invasion, metastasis, and apoptosis is well studied, stimulation or inhibition of beta adrenergic signaling can also have profound effects on cell metabolism. In normal cells, catecholamines promote lipolysis through beta adrenergic signaling [121], generating free fatty acids that can be metabolized through fatty acid (beta) oxidation (FAO), and conferring an alternative route for cellular energy production or the generation of essential metabolic intermediates [122]. Activation of β-ARs also increases the expression of the transcription co-factor, PGC-1α, and the transcription factors PPAR-α and PPAR-γ, which are involved in fatty acid oxidation and lipid processing. These factors form a central part of an adipogenic signature identified in hemangiosarcoma cell lines and primary hemangiosarcomas [54], suggesting part of the signaling pathways comprising this signature are driven by beta adrenergic signaling. The data also point to an essential metabolic role for FAO in hemangiosarcoma and angiosarcoma progression. Beta blockade by propranolol counters FAO by enhancing the synthesis of fatty acids, triacylglycerol, and phospholipids along with alterations in intracellular cholesterol trafficking [123], an effect noted in an angiosarcoma cell line treated with propranolol [54]. The increased expression of genes involved in fatty acid synthesis and cholesterol trafficking was also demonstrated in propranolol-treated IH endothelial cells [124], suggesting that propranolol promotes a switch away from catabolic pathways and toward anabolic pathways. We believe that it is this switch that is detrimental to angiosarcoma and hemangiosarcoma progression. Based on these data, we predicted that propranolol treatment should lead to changes in mitochondrial activity. Indeed, changes in mitochondrial biogenesis in angiosarcoma and hemangiosarcoma cell lines treated with propranolol were evident (Korpela and Dickerson, unpublished observation), supporting the induction of a metabolic switch by beta blockade. Studies are underway to better understand this process, exploit the potential metabolic Achilles heel of these tumors, and determine if beta blockade induces a similar metabolic switch in other sarcomas.

The observed adipogenic signature might also reflect the ability of hemangiosarcoma cells to alter the metabolism of normal cells within the TME. Tumorigenesis involves the constant communication between tumor cells and neighboring normal cells, creating an ecosystem of cancer cells competing or cooperating with other cells to promote tumor growth. Because cancer cells can impose a self-serving metabolic program on normal cells, these cells are recruited to supply substrates that provide energy to sustain tumor growth and promote angiogenesis, invasion, and metastasis [125]. Normal cells are also remodeled into cells such as cancer-associated fibroblasts (CAFs) and adipocytes (CAAs), which contribute to the composition of the TME and generate a niche favoring tumor growth [126,127]. Norepinephrine and epinephrine present within the TME may activate lipolysis in normal adipose cells and CAAs, leading to the release of free fatty acids. The released fatty acids could then be taken up by hemangiosarcoma cells and processed via FAO. Adipocytes might also produce inhibitory factors, such as adiponectin, and it is the balance of these factors that determine the participation of adipocytes in metabolic symbiosis and tumorigenesis. Therapeutic approaches aimed at reversing the cancer-imposed metabolic programming in the TME should include drugs that inhibit the cancer cell’s ability to seize the metabolism of stromal cells. Alternatively, drugs that interrupt the cancer cell’s capacity to use the energy resources produced by the CAFs or CAAs may also be of benefit.

## 5. Conclusions

An increasing body of knowledge from preclinical investigations and clinical retrospective analyses has shown that beta blockers have an immense potential in the treatment of human cancer patients with a wide range of tumor types. These findings emphasize the immediate need for retrospective analyses in veterinary oncology to determine the translational potential of these drugs in veterinary practice. The remarkable efficacy of beta blockers against benign vascular tumors and the translation of these findings toward vascular tumors of malignancy has allowed us to zero in on beta adrenergic signaling as a potential driver of angiosarcoma and hemangiosarcoma progression. Because well-characterized antagonists of β-ARs already exist and their effects in veterinary practice are well established, advancing the clinical use of these antagonists does not face the typical barriers associated with many newer drugs, and side effects from the inhibitors can be managed clinically by most veterinary hospitals. Therefore, beta blockade could provide an immediate adjunct therapy for dogs diagnosed with certain types of hemangiosarcoma. Continuing to explore the mechanistic actions of beta blockers on tumor cells, as well as their effect on the TME, will advance the use of these drugs in combination with standard of care treatments or with other interventions.
